# Decrease in Pneumococcal Co-Colonization following Vaccination with the Seven-Valent Pneumococcal Conjugate Vaccine

**DOI:** 10.1371/journal.pone.0030235

**Published:** 2012-01-12

**Authors:** Carina Valente, Jason Hinds, Francisco Pinto, Silvio D. Brugger, Katherine Gould, Kathrin Mühlemann, Hermínia de Lencastre, Raquel Sá-Leão

**Affiliations:** 1 Laboratory of Molecular Microbiology of Human Pathogens, Instituto de Tecnologia Química e Biológica, Universidade Nova de Lisboa, Oeiras, Portugal; 2 Laboratory of Molecular Genetics, Instituto de Tecnologia Química e Biológica, Universidade Nova de Lisboa, Oeiras, Portugal; 3 Bacterial Microarray Group, St. George's, University of London, London, United Kingdom; 4 Centro de Química e Bioquímica, Departamento de Química e Bioquímica, Faculdade de Ciências da Universidade de Lisboa, Lisbon, Portugal; 5 Institute for Infectious Diseases, University of Bern, Bern, Switzerland; 6 Laboratory of Microbiology, The Rockefeller University, New York, New York, United States of America; Instituto Butantan, Brazil

## Abstract

Understanding the epidemiology of pneumococcal co-colonization is important for monitoring vaccine effectiveness and the occurrence of horizontal gene transfer between pneumococcal strains. In this study we aimed to evaluate the impact of the seven-valent pneumococcal conjugate vaccine (PCV7) on pneumococcal co-colonization among Portuguese children. Nasopharyngeal samples from children up to 6 years old yielding a pneumococcal culture were clustered into three groups: pre-vaccine era (n = 173), unvaccinated children of the vaccine era (n = 169), and fully vaccinated children (4 doses; n = 150). Co-colonization, serotype identification, and relative serotype abundance were detected by analysis of DNA of the total bacterial growth of the primary culture plate using the *ply*NCR-RFLP method and a molecular serotyping microarray-based strategy. The *ply*NCR-RFLP method detected an overall co-colonization rate of 20.1%. Microarray analysis confirmed the *ply*NCR-RFLP results. Vaccination status was the only factor found to be significantly associated with co-colonization: co-colonization rates were significantly lower (p = 0.004; Fisher's exact test) among fully vaccinated children (8.0%) than among children from the pre-PCV7 era (17.3%) or unvaccinated children of the PCV7 era (18.3%). In the PCV7 era there were significantly less non-vaccine type (NVT) co-colonization events than would be expected based on the NVT distribution observed in the pre-PCV7 era (p = 0.024). In conclusion, vaccination with PCV7 resulted in a lower co-colonization rate due to an asymmetric distribution between NVTs found in single and co-colonized samples. We propose that some NVTs prevalent in the PCV7 era are more competitive than others, hampering their co-existence in the same niche. This result may have important implications since a decrease in co-colonization events is expected to translate in decreased opportunities for horizontal gene transfer, hindering pneumococcal evolution events such as acquisition of antibiotic resistance determinants or capsular switch. This might represent a novel potential benefit of conjugate vaccines.

## Introduction

*Streptococcus pneumoniae* (the pneumococcus) remains a main cause of morbidity and mortality worldwide [1]. Its ecological niche is the human nasopharynx. Colonization by pneumococcus can occur soon after birth and remains high in the first years of life [2]. Virtually every child is colonized by pneumococcus at some stage in life and each serotype can colonize for several weeks being then replaced by another serotype or reacquired [3], [4].

Although poorly studied, it has been known for decades that simultaneous carriage of multiple pneumococci (or co-colonization) can occur [5], [6]. Co-colonization is an important event for pneumococcal evolution as it represents an opportunity for horizontal gene transfer, the main mechanism of evolution in this species [7], [8].

Studies on co-colonization have been hampered by the lack of suitable detection methods. The limited reports available have found co-colonization rates in the range of 5–30% [3], [9], [10], [11], [12], [13], [14]. However, most studies have relied on serotyping individual colonies isolated from culture. This approach has low sensitivity, is expensive and time-consuming and is biased to detect only the most abundant serotypes [10], [15].

In recent years, with the introduction of multivalent pneumococcal conjugate vaccines, there has been a renewed interest in the study of co-colonization since it is important to understand serotype changes among carriers following vaccination, for instance, to distinguish increased acquisition from unmasking phenomena [16].

Simultaneously, novel approaches for detection of co-colonization have been proposed [9], [13], [17], [18], [19]. In particular, Brugger *et. al*. have developed, the *ply*NCR-RFLP method, based on the restriction pattern of a highly conserved DNA region within the pneumococcal species [9]. Additionally, Hinds *et al.* developed a molecular serotyping microarray based on genomic DNA hybridization that is able to detect and quantify all serotypes described to date [20].

In Portugal, the seven-valent pneumococcal conjugate vaccine (PCV7) became commercially available in June 2001 and, though the vaccine is not included in the National Immunization Program, it has been widely prescribed as the Portuguese Society of Pediatrics issued recommendation for PCV use among all children up to five years of age. These included a catch up schedule. Rates of PCV coverage increased gradually since 2001. Estimates from Pfizer based on annual sales and considering 3.5 doses per newborn are as follows from 2001 to 2007: 17%, 32%, 56%, 65%, 63%, 75%, and 79%, respectively. In studies conducted by us, by 2006–2007, c.a. 70% of children (aged up to 6 years old) had received at least one PCV7 dose [21]. Major serotype shifts have occurred since 2001 both in disease and colonization [22], [23]. Although colonization rates have remained stable [22], [23], the effect of vaccination on co-colonization has remained unknown.

In this study, using a combination of the *ply*NCR-RFLP and the molecular serotyping microarray methods, we aimed to gain insights on the prevalence of co-colonization and evaluate potential changes that might have occurred following vaccination with PCV7 in Portugal.

## Results

### Co-colonization detected by colony morphology and by *ply*NCR-RFLP

Of the 492 nasopharyngeal samples studied, two colonies with distinct morphologies were identified in the primary selective agar plate of 32 samples. Among these, two serotypes were isolated in 17 samples; the remaining samples yielded a single serotype. Thus, the co-colonization rate detected by this method was 3.5%. The *ply*NCR-RFLP method detected pneumococcal co-colonization in 20.1% of the 492 samples (n = 99), a significantly higher proportion than the one found with the former approach (p<0.001, X^2^ test).

### Confirmation of co-colonization by molecular serotyping microarray

A total of 165 samples were blindly tested using the microarray: the 99 samples in which pneumococcal co-colonization was detected by the *ply*NCR-RFLP, and 66 samples for which there was no evidence of co-colonization. Microarray results were in agreement with those generated by the *ply*NCR-RFLP method.

In addition to identifying the serotypes present in samples, the microarray was also able to detect the presence of non-encapsulated pneumococci and/or closely related *Streptococcus* spp., collectively called non-typeables (NT). These were detected in 40 samples, all of which had an indication of co-colonization by the *ply*NCR-RFLP method. However, as both methods did not confidently discriminate between NT species within co-colonized samples, we decided to exclude all NT from further analysis to avoid possible over-reporting of true pneumococcal co-colonization. Hence, all results presented in this report involve only pneumococci for which serotypes were identified. Still, when a separate analysis was performed, assuming that all NT were *bona fide* non-encapsulated pneumococci, the same conclusions described below were obtained (data not shown).

Upon exclusion of all NTs, the microarray detected more than one pneumococcal serotype (i.e. more than one capsulated strain) in 73 samples that had an indication of co-colonization.

### Confirmation of serotypes detected by the microarray

All serotypes identified by the microarray were confirmed by PCR using as template purified DNA of the primary selective growth. New primers, targeting specific capsule biosynthetic genes or variants not covered by the CDC scheme, were designed as needed (Table S1).

### Factors associated with pneumococcal co-colonization

**Figure 1:**
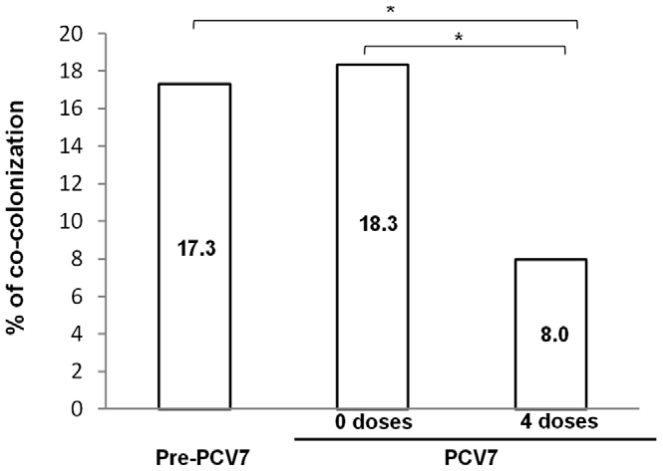
Frequency of co-colonization in the three study groups.

Vaccination status and attendance of day-care center F were the only variables significantly associated with co-colonization and both were protective factors (Table 1). Vaccinated children presented significantly lower (p = 0.004; Fisher's exact test) co-colonization rates (8.0%) than non-vaccinated children (regardless of whether the latter were from the pre-PCV7 era (17.3%) or from the PCV7 era (18.3%)) (Figure 1).

**Table 1:** Risk factors for pneumococcal co-colonization in univariate and multivariate analysis.

Characteristic	No. ofcarriers	No. (%) ofco-colonized carriers	Univariate analysis^c^		Multivariate analysis^d^	
			OR (95% CI)	*p*	OR (95% CI)	*p*
Age (in months)	(-)^f^	(-)^f^	(-)^f^	0.59^e^	0.99 [0.97–1.02]	0.60
Sex						
Male	255	41 (16.1%)	1.23 [0.74–2.02]	0.45	1.18 [0.70–1.99]	0.54
Female	237	32 (13.5%)	Reference		Reference	
Day-care center attended (out of 15)			(-)^f^	0.15^g^		
Unit F^h^	74	4 (5.4%)	**0.29 [0.10–0.82]**	**0.01**	**0.15 [0.04–0.56]**	**0.005**
Other units	418	69 (16.5%)	Reference		Reference	
Time period						
Pre-PCV7	173	30 (17.3%)	0.74 [0.45–1.23]	0.29	1.73 [0.82–3.66]	0.15
PCV7	319	43 (13.5%)	Reference		Reference	
Vaccination status						
Unvaccinated^i^	342	61 (17.8%)	**0.40 [0.21–0.77]**	**0.004**	**0.34 [0.16–0.72]**	**0.005**
Vaccinated (4 doses)	150	12 (8.0%)	Reference		Reference	

c Fisher's exact test (Monte Carlo estimation with 10,000 simulations), except when indicated.

d Multivariate logistic regression.

e Kolmogorov-Smirnov test.

f not available for variables that are continuous or have more than two classes.

g test applied to all day-care centers simultaneously.

h results are only shown for unit F as the results for other units were not significant.

i includes unvaccinated children from pre-PCV7 and PCV7 era.

### Co-colonization patterns

Among the 73 co-colonized samples (excluding NT), the microarray identified two serotypes in 59 samples, three serotypes in 13 samples, and six serotypes in one sample (Table 2). When each type of co-colonization (double, triple, and sextuple) was analyzed separately, a higher proportion of each type of co-colonization was still noted among the unvaccinated children. However, probably due to the low number of observations, the results did not reach statistical significance (Table 2).

**Table 2:** Colonization events according to study group.

No. of serotypes detected in samples	Pre-PCV7 era, n = 173(%)	PCV7 era		Total, n = 492(%)	*p* value
		Unvaccinated, n = 169(%)	Vaccinated, n = 150(%)		
1	143 (82.6)	138 (81.7)	138 (92.0)	419 (85.2)	0.018^a^
>1(all co-colonization samples)	30 (17.3)	31 (18.3)	12 (8.0)	73 (14.8)	0.018^a^
2	25 (14.4)	23 (13.6)	11 (7.3)	59 (12.0)	0.11^a^
3	4 (2.3)	8 (4.7)	1 (0.7)	13 (2.6)	0.08^b^
4	0 (-)	0 (-)	0 (-)	0 (-)	-
5	0 (-)	0 (-)	0 (-)	0 (-)	-
6	1 (0.6)	0 (-)	0 (-)	1 (0.2)	0.65^b^

a X^2^ test.

b Fisher's exact test.

### Serotype distribution

**Figure 2:**
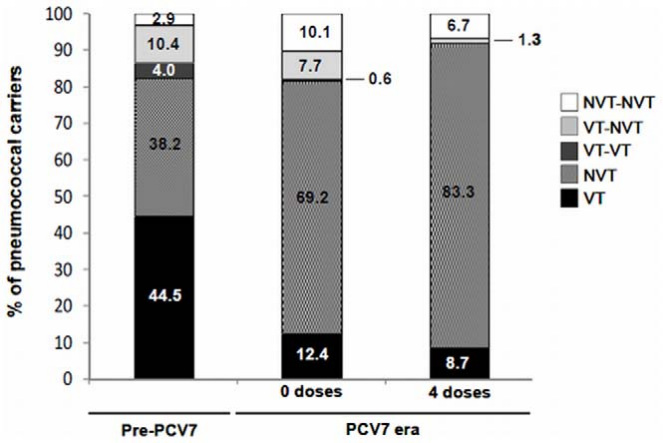
Single and co-colonization events by vaccine-types (VT) and non-vaccine types (NVT) in the three study groups. *Statistically significant differences (p* = *0.004; Fisher's exact test).

In line with previous observations, a significant serotype replacement effect (of vaccine types (VTs) by non-vaccine types (NVTs)) was observed among samples from the PCV7 era compared to samples from the pre-PCV7 era (Figure 2). This effect was noted both in single and co-colonization events and among vaccinated and non-vaccinated children, although it was more pronounced in the former group.

When the relative proportion of VTs in single and co-colonization in the pre-PCV7 and PCV7 eras were compared, no significant changes were observed (p = 0.338, Fisher exact test). However, this was not true for NVTs: in the PCV7 era there were significantly less NVT co-colonization events than would be expected based on the NVT distribution observed in the pre-PCV7 era (p = 0.024). This result, suggested that, in the PCV7 era, there was an asymmetric distribution among NVTs found in single or co-colonizing events. Such effect was more pronounced among vaccinated children (where serotype replacement effect was strongest) leading to a decreased rate of co-colonization among this group.

**Figure 3:**
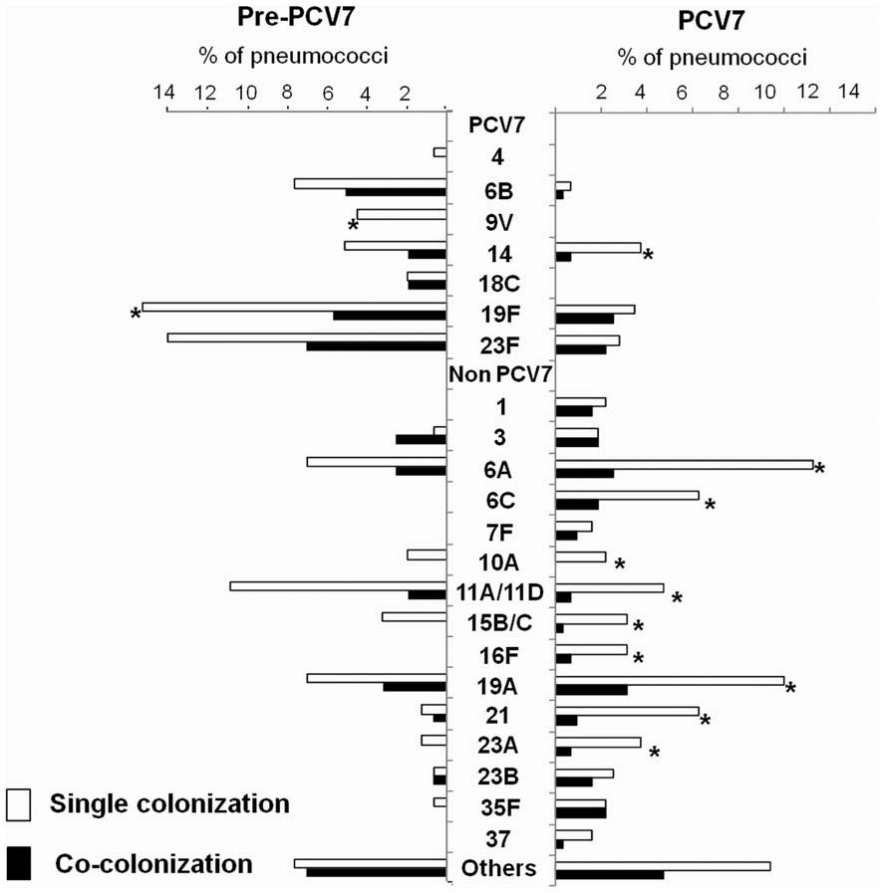
Individual serotype distribution of single and co-colonized samples from the pre-PCV7 and PCV7 eras. Non-vaccine serotypes with an absolute frequency <5 in the PCV7 era were grouped as “others”. These include serotypes 8, 9L, 9N, 13, 15A, 17F, 18A, 18F, 19B, 19C, 22F, 24F, 29/35B, 31, 33A/33F, 34, 38, 42, 33B-like, 35A/C, 36/15B-like, and 39-lik. *Observed frequency in co-colonization is significantly lower than expected.

Furthermore, the distribution of individual serotypes found in co-colonized samples reflected, in general, the distribution found in samples containing a single strain, i.e., serotypes frequent in single colonization tended to be also frequent in co-colonization (Figure 3). Serotypes 19F and 23F were the most frequent colonizers of the nasopharynx in the pre-PCV7 era and serotypes 6A and 19A dominated in the PCV7 era.

### Relative abundance of serotypes of co-colonized samples

For each co-colonization event the relative proportion of each serotype was determined by the microarray. We observed that there was a clear dominance of one serotype over the other(s) in a sample in 71.9% the cases involving associations of NVTs. In the associations of two or more VTs, or VTs with NVTs, this dominance was not so evident being 62.5% and 42.4%, respectively.

To investigate if individual serotypes had been detected in co-colonization at a frequency significantly different from the one expected by chance alone, a permutation analysis was done for each time period. Serotypes 9V and 19F (in the pre-PCV7 era), and 6A, 6C, 10A, 11A/11D, 14, 15B/C, 16F, 19A, 21, and 23A (in the PCV7 era) were found less frequently in co-colonization than expected (Figure 3). A more detailed analysis aimed to detect specific associations between pairs of serotypes was not possible due to the low numbers of each pair.

## Discussion

We aimed to study the impact of PCV7 on pneumococcal co-colonization rates by combining two recently developed methods, *ply*-NCR RFLP and molecular serotyping microarray, which enabled detection of co-colonization and the identification and relative quantification of the serotypes present in those samples.

After excluding NT species, a co-colonization rate by capsulated pneumococci of approximately 15%, confirmed by both the *ply*NCR-RFLP and microarray methods was observed. The combination of these two methods allowed four times more sensitivity in detection of co-colonization than serotyping of individual colonies exhibiting distinct morphologies.

Our results show that introduction of PCV7 led to a serotype replacement effect among vaccinated children and, although less pronounced, also among non-vaccinated children. As a result, in the PCV7 era, a decrease in the proportion of VTs was observed in single and co-colonized carriers of both groups. These findings are in line with previous observations that indicated that, in settings where vaccination rates are high, vaccination reduces not only the proportion of vaccinated individuals colonized with VTs but also among non-vaccinated individuals in the community due to an indirect effect resulting from decreased transmission of VTs [24].

Interestingly, co-colonization rates were significantly lower among vaccinated children (8.0%) than among non-vaccinated children (c.a. 18%, irrespective of whether they were from the pre-PCV7 or PCV7 eras). Of the variables studied, attendance of day-care center F and, more importantly, vaccination status were the only factors that accounted for these phenomena.

Comparison of the characteristics of day-care center F with the other 14 day-care units did not reveal obvious differences between them (in carriage prevalence, area, number of children, crowding, or antimicrobial consumption). However, this center was enriched on serotypes (6A, 11A/D, and 19A) known to be less associated with co-colonization than expected by chance alone (as discussed below) which, per se, could potentially explain this observation.

The finding that co-colonization was diminished among PCV7-vaccinated children is of particular interest as it may have important implications for pneumococcal evolution. Indeed, decreased co-colonization is expected to translate in fewer opportunities for horizontal gene transfer hindering, for example, transference of resistance genes and emergence of vaccine escape recombinants.

But how to explain such observations? A closer inspection of Figure 2 indicates that among vaccinated children a significant reduction of VTs led to an asymmetric redistribution of the proportion of single and multiple NVT carriers. Specifically, it resulted in a higher than expected proportion of single NVT carriers and a lower than expected proportion of NVT/NVT multiple carriers.

One possible interpretation for the latter observation is that some NVTs in circulation in the PCV7 era are more competitive than others, impairing their co-existence in the same ecological niche. Evidence for competition between serotypes in the nasopharynx was described before and mechanisms leading to it (such as bacteriocin production) have been identified [25], [26]. Two observations in the study support this hypothesis: (i) the fact that in the association of two or more NVTs, there was a clear dominance of one serotype in 71% of the cases, while such high values were not observed in the association of two or more VTs (63%) or in the association of VTs with NVTs (42%); and (ii) in the PCV7 era, there were a number of NVTs that were found less frequently in co-colonization than would be predicted by chance alone. These serotypes were among the group of the most prevalent ones in single colonization for the same period suggesting they are highly competitive.

Our results contrast with those from a recent study in Switzerland that reported a stable rate of co-colonization upon introduction of PCV7 and a balanced co-existence of serotypes found in co-colonized samples [27]. That study, however, differs from ours in several aspects hindering comparisons between findings. The study population consisted of patients with acute otitis media or pneumonia (and hence, not healthy), children and adults were included, colonization rates were significantly lower (even when the same age groups were compared), and the starting material was the swab without a culturing step. Furthermore, if competition between serotypes is indeed relevant, local serotype distribution may further affect the results obtained. Additional studies are warranted to explore this subject.

Of note, we observed that in our setting (as in many others), in the vaccine era, serotypes 6A and 19A were the most frequent colonizers of the nasopharynx in single and co-colonization. As the recently introduced PCV13 includes these two serotypes, it will be important to monitor how co-colonization is affected by it.

Our study has some limitations. Firstly, it is a cross-sectional study and therefore duration of carriage, known to vary according to the serotype, was not taken into account [3]. This has at least two implications: on one hand, the co-colonization rate may have been underestimated as serotypes with shorter duration of carriage may have been missed; on the other hand, we were unable to determine whether the co-colonized samples result from a truly co-existence colonization event or merely reflect a transitional state between serotypes colonizing the nasopharynx.

Secondly, the direct culture of the nasopharyngeal swab has some disadvantages. As with any culturing step, the chance of occurrence of sample contamination during handling increases. In addition, variation in the inoculum size may have occurred. This could have been partially overcome if the swab would have been first diluted in a liquid medium and, afterwards, a fixed amount of the liquid sample plated. Also, the use of a cultivation step and the choice of a selective (gentamycin blood agar) medium may have altered the composition of the sample. Finally, the culturing step may lead to false-negative results (as we analyzed viable cells only). Still, despite these limitations, our strategy has the advantage of increasing the amount of pneumococcal DNA in the samples. Direct analysis of the swabs often leads to limited amounts of DNA hindering further processing of the sample; for example in a previous study using the *ply*NCR-RFLP method, 21% of the samples did not yield sufficient DNA for analysis [9].

The third limitation is the current inability of the microarray to discriminate between non-encapsulated pneumococci from other closely related *Streptococcus* spp. such as *S. mitis* when within a complex mixture containing other pneumococci. In this study, we ignored the presence of these NT organisms but a separate analysis taking into account these results mimicked the general observations reported here. However, co-colonization with non-encapsulated pneumococcus or closely related species is of interest as this represents the wider gene pool available for horizontal gene transfer.

Finally, the inclusion of older children (up to 71 months) might be considered a limitation as it is not clear whether conjugate vaccines administered in the first and second year of life will have an effect on colonization 3–4 years afterwards. Still, a previous study by Millar *et al.* on the long-term effect of PCV in a community using a 3+1 schedule, found a significantly lower prevalence of VT carriage 27 months after vaccination when compared with control communities [28], suggesting there might be a prolonged effect of PCV7 on colonization. Furthermore, if we consider the opposite scenario, that is, that children of older ages are no longer protected by PCV7 then we would expect this group to have higher carriage of VTs, which would lead to a net increase in VT among the vaccinated group. This, in turn, according to our data, would result in a global increase in co-colonization among this group. In other words, the results presented by us for the vaccinated group would have a bias towards an increasing co-colonization rate. Still, the rate observed was significantly lower than among non-vaccinated children supporting our observations and suggesting that, if anything, the exclusion of older children would result in a even stronger disparity between co-colonization rates among vaccinated and unvaccinated children.

Our study has also some strength. The collection of samples used was well defined and matched and the methods used have high sensitivity. This allowed us to take some important conclusions in the context of co-colonization. Finally, we are convinced that, to our knowledge, this is one of the very few studies to date focusing on the impact of PCV7 on pneumococcal multiple carriage and is the first to report a significant difference between co-colonization rates of vaccinated and non-vaccinated children.

In summary, this study suggests that PCV7-vaccinated children have lower rates of pneumococcal co-colonization, resulting in decreased opportunities for horizontal gene transfer between strains. This represents a novel potential benefit of multivalent pneumococcal conjugate vaccines. Considering that these results may depend on local epidemiological factors and that serotype redistribution is occurring as novel vaccines are being introduced, additional studies are warranted to verify if similar results are obtained.

## Materials and Methods

### Study design

Nasopharyngeal (NP) swabs collected from healthy children attending day-care centers in Oeiras, Portugal, were retrospectively selected [23], [29]. In each year, samples were collected in the winter months of January to March. Samples were selected according to the following criteria: (i) swabs were obtained from children aged 18–71 months; (ii) children had not received antibiotic within the month preceding sampling; and (iii) swabs yielded a pneumococcal positive culture upon direct plating within four hours of collection as described below.

The samples were clustered in three groups matched for age and gender: group I included samples from the pre-vaccine era (n = 173, collected in 2001); group II included samples collected in the vaccine era from unvaccinated children (n = 169, collected in 2006–2007); and group III included samples collected in the vaccine era from fully vaccinated children (i.e., 4 doses, n = 150, collected in 2006–2007) (Table S2). This study was nested in a previous one aimed to study the impact of PCV7 on colonization [23]. Approval for the initial study was obtained from the Ministry of Education and directors of day-care centers. Signed informed consent was obtained from the parents or guardians of participating children. Samples and questionnaires were attributed a number and all information was treated anonymously.

### Isolation of pneumococci and sample preservation

NP samples were collected by pediatric nurses using mini-tip calcium alginate sterile swabs and inoculated directly within 4 hours in a primary selective plate of 5% blood trypticase soy agar containing gentamicin (5 mg/liter) to select for *S. pneumoniae*. Plates were incubated overnight at 37°C under anaerobic conditions, with an optochin disk. On the following day, presumptive pneumococcal colonies exhibiting different morphologies were picked (one colony per morphology) and subcultured. The remaining bacterial lawn was collected and frozen at −80°C in 1ml Mueller-Hinton broth containing 30% glycerol. On the third day, cultures derived from the isolated colonies were also frozen. Swabs from 2001 were discarded after plating; swabs from 2006 and 2007 were stored frozen.

### PCR serotyping

Pure cultures were serotyped by PCR, as described previously [30], using primers and conditions available at http://www.cdc.gov/ncidod/biotech/strep/pcr.htm. Strains that could not be typed by PCR were serotyped by the Quellung reaction using commercially available pneumococcal antisera (Statens Serum Institute, Copenhagen, Denmark).

### DNA isolation

Total DNA was isolated from 200 µl of the primary selective culture frozen stock using the High Pure PCR Template Preparation Kit (Roche Applied Science) according to the manufacturer's instructions.

### *ply*NCR-RFLP

Detection of co-colonization was done as previously described [9]. Briefly, the noncoding region between the pneumolysin gene and the preceding hypothetical protein gene was amplified by PCR. The product was separately digested with up to four restriction enzymes: AflIII, ApoI, DdeI, and MseI. A sample was assumed to contain more than one strain whenever the sum of the size of the digestion fragments was higher than the size of the undigested PCR product.

### Molecular serotyping microarray

The BµG@S SP-CPSv1.4.0 microarray designed for *S. pneumoniae* molecular serotyping was used following standard protocols previously described [27]. Microarray data was statistically analyzed using a Bayesian hierarchical model to determine the serotype, or combination of serotypes, present in the sample and assign their relative abundance [31]. In the interpretation of co-colonization results, a serotype was classified as dominant if its relative abundance in the sample was ≥70%.

### Statistical analysis

Pair-wise associations between co-colonization and vaccination status, time period, age, gender and day-care center were measured. Statistical significance was accessed through Fisher exact tests. A Kolmogorov-Smirnov test was applied to compare age distribution between multiple and single carriers. Associations were considered significant if p<0.05.

Multivariate logistic regression models were used to detect associations with co-colonization, using vaccination status, time period, age, gender and day-care center as independent variables. As day-care center was a categorical variable with more than two categories, 14 dummy variables (binary variables for all but one day-care center) were constructed and used.

Models were adjusted with all independent variables simultaneously and through backward and forward stepwise variable selection methods (which produced the same final models). Models were adjusted to subsets of data including only vaccine period individuals, or excluding vaccinated individuals. Although only 73 of 492 samples were positive for co-colonization (as detailed in the results' section) all 492 samples were used in the multiple logistic regression enabling the analyses of all variables under study. Indeed, according to Peduzzi et al. [32], the size of our sample allows models with up to 7–8 predictor variables. Furthermore, according to the later work of Vittinghoff and McCulloch [33], this number of predictors can safely be double if the aim of the analysis is to detect associations in observational data and not making predictions; or if the high number of predictors is necessary to control possible confounding variables. Our study fitted both conditions.

Permutation analysis was done to test if serotypes were found in co-colonized carriers at frequencies different than expected by chance. For each time period, serotype identifications were randomly allocated to children (including not colonized) 2000 times. Serotype frequencies were maintained. The p-values obtained for all serotypes were corrected for multiple testing by controlling the False Discovery Rate below 0.05 [34].

Analyses were performed in SPSS 17.0 and in Matlab 7.7.

## Supporting Information

Table S1 **List of oligonucleotides designed for this study.**
(DOCX)

Table S2 **Age distribution in the three groups.** Comparison of age distribution in the three groups was done by a Kolmogorov-Smirnov test using the age of each child in months. The results were not significantly different (p = 0.614).
(DOCX)
