# Supplementary material for: Decrease in Pneumococcal Co-Colonization following Vaccination with the Seven-Valent Pneumococcal Conjugate Vaccine
Source: PLoS One. 2012 Jan 12;7(1):e30235. doi: 10.1371/journal.pone.0030235 (PMC3257259; doi:10.1371/journal.pone.0030235)
Supplement: Table S1 — List of oligonucleotides designed for this study. (DOCX) [file pone.0030235.s001.docx]

| Primer name | Serotype targeted | Gene | Fragment size (bp) | Primer sequence (5’-3’) |
| --- | --- | --- | --- | --- |
| wchL_15B_F | 15B | *wchL* | 449 | 5’-gtgattttggtgaatgatgggtctaacgattct-3’ |
| wchL_15B_R |  |  |  | 5’-gacatatcttcacctagtgacatctcagtcg-3’ |
| wcxR_16A_F | 16A | *wcxR* | 387 | 5’-gcccctagaaaagttcctgctagcatcacg-3’ |
| wcxR_16A_R |  |  |  | 5’-gtggataggggacaacgatag-3’ |
| wzy_19B/C_F | 19B/C | *wzy* | 375 | 5’-ccaagtaaatgctctaattccgagag-3’ |
| wzy_19B/C_R |  |  |  | 5’-gcattcgtttatggaggtggattgg-3’ |
| wchU_19C_F | 19C | *wchU* | 371 | 5’-gcccccacataatgaactttttcaactgg-3’ |
| wchU_19C_R |  |  |  | 5’-gctctttgctatggtttaccatgtatcgg-3’ |
| wzy_27_F | 27 | *wzy* | 617 | 5’-gctctcccatgcatgcttgcaggatttagagc-3’ |
| wzy_27_R |  |  |  | 5’-gacactactcctaaggaaaagagggaggctatacc-3’ |
| wciO_33B_F | 33B | *wcjO* | 245 | 5’-ggtagacctctatcatacatattgacaattcc-3’ |
| wciO_33B_R |  |  |  | 5’-gcatctataacacttcccataggagaggtaattccc-3’ |
| wcjH_35F_F | 35F | *wcjH* | 295 | 5’-cagcatctttatacatgctccg-3’ |
| wcjH_35F_R |  |  |  | 5’-ctgtgaaacctggtttgc-3’ |
| wcjA_36_F | 36 | *wcjA* | 622 | 5’-ggtggcgctgagaggattgtgtaccag-3’ |
| wcjA_36_R |  |  |  | 5’-cagttctaagctctccatcaccaac-3’ |
| wcrD _39_F | 39 | *wcrD* | 562 | 5’-ggtggacatgggtcaacgattaatgttggt-3’ |
| wcrD _39_R |  |  |  | 5’-agtatgtcaatacgtctctgactactgg-3’ |
