# Supplementary material for: Decrease in Pneumococcal Co-Colonization following Vaccination with the Seven-Valent Pneumococcal Conjugate Vaccine
Source: PLoS One. 2012 Jan 12;7(1):e30235. doi: 10.1371/journal.pone.0030235 (PMC3257259; doi:10.1371/journal.pone.0030235)
Supplement: Table S2 — Age distribution in the three groups. Comparison of age distribution in the three groups was done by a Kolmogorov-Smirnov test using the age of each child in months. The results were not significantly different (p = 0.614). (DOCX) [file pone.0030235.s002.docx]

| Age (yr) | No. of children per group (%) | | |
| --- | --- | --- | --- |
|  | Pre-PCV7 period | PCV7 period  (0 doses) | PCV7 period  (4 doses) |
| <2 | 15 (8.7) | 4 (2.4) | 11 (7.3) |
| 2-<4 | 50 (28.9) | 58 (34.3) | 63 (42.0) |
| 4-<6 | 108 (62.4) | 107 (63.3) | 76 (50.7) |
| Total: | 173 | 169 | 150 |
